# Self-management skills in adolescents with chronic rheumatic disease: A cross-sectional survey

**DOI:** 10.1186/1546-0096-9-35

**Published:** 2011-12-06

**Authors:** Erica F Lawson, Aimee O Hersh, Mark A Applebaum, Edward H Yelin, Megumi J Okumura, Emily von Scheven

**Affiliations:** 1 Department of Pediatrics, Division of Rheumatology & Immunology, University of California, San Francisco, 533 Parnassus, Rm U-127, Box 0107, San Francisco, CA 94143, USA; 2 Department of Pediatrics, Division of Allergy, Immunology & Rheumatology, University of Utah, 295 Chipeta Way, Salt Lake City, UT 84108, USA; 3 Department of Medicine, Division of Rheumatology, University of California, San Francisco, 3333 California Street, Suite 270, San Francisco, CA 94143-0920, USA; 4 Department of Pediatrics, Division of General Pediatrics, University of California, San Francisco, 3333 California Street, Suite 245, San Francisco, CA 94118, USA

**Keywords:** Self-management, transition, pediatric rheumatology

## Abstract

**Background:**

For adolescents with a diagnosis of lifelong chronic illness, mastery of self-management skills is a critical component of the transition to adult care. This study aims to examine self-reported medication adherence and self-care skills among adolescents with chronic rheumatic disease.

**Methods:**

Cross-sectional survey of 52 adolescent patients in the Pediatric Rheumatology Clinic at UCSF. Outcome measures were self-reported medication adherence, medication regimen knowledge and independence in health care tasks. Predictors of self-management included age, disease perception, self-care agency, demographics and self-reported health status. Bivariate associations were assessed using the Student's t-test, Wilcoxon rank sum test and Fisher exact test as appropriate. Independence in self-management tasks were compared between subjects age 13-16 and 17-20 using the chi-squared test.

**Results:**

Subjects were age 13-20 years (mean 15.9); 79% were female. Diagnoses included juvenile idiopathic arthritis (44%), lupus (35%), and other rheumatic conditions (21%). Mean disease duration was 5.3 years (SD 4.0). Fifty four percent reported perfect adherence to medications, 40% reported 1-2 missed doses per week, and 6% reported missing 3 or more doses. The most common reason for missing medications was forgetfulness. Among health care tasks, there was an age-related increase in ability to fill prescriptions, schedule appointments, arrange transportation, ask questions of doctors, manage insurance, and recognize symptoms of illness. Ability to take medications as directed, keep a calendar of appointments, and maintain a personal medical file did not improve with age.

**Conclusions:**

This study suggests that adolescents with chronic rheumatic disease may need additional support to achieve independence in self-management.

## Background

Children with special health care needs (CSHCN), which include children with chronic rheumatologic conditions, typically enter the health care system as passive participants, with parents assuming responsibility for the majority of their health care-related tasks. However, as pediatric patients age into adolescence and young adulthood, there is an expected shift in the burden of responsibility from parent to young adult. Young adults must learn to complete such tasks as scheduling appointments, communicating with providers, securing insurance coverage, and taking medications as directed. Prior studies have shown that CSHCN are often unprepared to make this transition [1,2], leaving them at risk for ongoing dependence on their families and delayed achievement of developmental milestones [3]. While the American Academy of Pediatrics has recommended transition planning for all CSHCN since the 1980s, only 41% of CSHCN receive transition-planning services [4]. Among young people with juvenile idiopathic arthritis (JIA), studies have shown high rates of unsuccessful transfer to adult care [5]. Many studies have assessed the challenges that CSHCN face during transition, but evidence-based recommendations to improve transitional care for CSHCN are lacking, and even less is known about the optimal management of rheumatologic disease-specific issues related to transitioning.

Self-management has been defined as an "individual's ability to manage the symptoms, treatment, physical and psychosocial consequences and lifestyle changes inherent in living with a chronic condition" [6]. The development of self-management skills is a critical part of the transition to adulthood for adolescents with a diagnosis of lifelong chronic illness, and failure to develop these skills can prevent successful transition to adult care. Difficulties with self-management in late adolescence and young adulthood may be exacerbated by the transition to new providers and changes in insurance. In most of the United States, public health plans such as Medicaid, SCHIP and the federal Title V program facilitate access to comprehensive health care for CSHCN through age 18 or 21, at which point patients typically "age out" of coverage [7]. Following loss of insurance patients often experience gaps in care, with lapses in doctor visits and medication adherence placing them at risk for increased disease activity and adverse health outcomes [8]. Among patients with rheumatic diseases such as systemic lupus erythematosus and juvenile idiopathic arthritis, ongoing disease activity increases the risk of permanent damage and functional limitation [9]. The problem of poor outcomes in the transition period has been demonstrated in multiple populations [1,10].

Little is known about the self-management skills of adolescents with chronic rheumatic disease, though a recent study showed improved disease knowledge and decreased pain among subjects receiving an internet-based intervention designed to improve self-management in JIA [11]. It is unclear which health care tasks adolescents are completing independently and which tasks they feel unable to complete. While prevalence and causes of medication non-adherence are also not well understood, non-adherence has been associated with poorer health perceptions, self-esteem, mental health, family cohesion, and social functioning [12]. It is unclear whether health-related self-management skills are improving with increasing age, as patients prepare to transfer to adult rheumatology care. Therefore, this cross-sectional study examined self-reported adherence and health-care-related behaviors, as a first step toward assessing health-related self-management among adolescents with rheumatic disease prior to transfer to adult-centered care.

## Methods

### Subjects and Data Collection

We recruited 52 consecutive patients cared for in the Pediatric Rheumatology Clinic at the University of California, San Francisco (UCSF) between February and April of 2009. The UCSF Pediatric Rheumatology Clinic has a wide referral base, caring for patients with a spectrum of rheumatic diseases throughout Northern California. In 2009 there were approximately 1600 outpatient clinic visits. Patients are cared for up to age 21, at which point they are transferred to adult providers, either at UCSF or in the community.

Criteria for inclusion included age 13 through 20 years, and a confirmed diagnosis of chronic rheumatic disease that would require transition to an adult specialist. Exclusion criteria were inability to speak or read English and cognitive impairment that would prevent future independence in health care management. No compensation for participation was provided. During the recruitment period, 16 patients who met inclusion and exclusion criteria failed to present for a scheduled appointment.

Patients were recruited from all clinic sessions following a convenience sampling strategy. Informed consent was obtained using a protocol approved by the University of California, San Francisco Committee on Human Research. Completion of the visit took approximately 30 minutes and the majority of patients approached agreed to participate. During the recruitment period 87 patients who met inclusion criteria were seen in clinic and 60% were enrolled. Participants completed a transition-readiness survey that included multiple choice, Likert scale, and free-response questions [13-16]. Subjects completed the surveys at a single clinic visit.

### Outcome Measures

Disease self-management was assessed in two domains: independence in self-management tasks and self-reported medication adherence. Independence in self-management tasks was assessed with a 15-question tool, which was adapted from the California Healthy and Ready to Work Health Care Transition Guide [13]. Subjects were asked to report whether a given health care task was typically completed independently, completed with some assistance, or completed by someone else. Subjects were considered proficient if they could complete a task without any assistance. Health care tasks included carrying an insurance card, refilling prescriptions, and keeping a calendar of appointments. Medication adherence was assessed with 3 multiple-choice questions developed by the investigators. The first asked whether doses of medication were missed. The second assessed frequency of missed doses. The third asked why subjects missed doses. Subjects were defined as adherent if they reported taking all doses of their medication in a typical week.

### Predictors of Self-Management

Predictors of interest included medication regimen knowledge, disease perception, self-care agency, demographics, and health status. To assess medication regimen concordance, patients were asked to provide the name, dosing regimen, and purpose for each of their medications. This patient-generated list was compared to the medication list in the clinic note from the day the survey was administered. Concordance for medication names, dosing intervals, and purposes were separately calculated as the percent of correct responses. For example, if a patient was prescribed 3 medications and listed the correct name for 3, the correct dosing interval for 2, and the correct purpose for 1, then concordance for name, dosing interval and purpose would be calculated as 100%, 67% and 33%, respectively. In assessing concordance for medication purpose, responses were scored as correct if answers demonstrated any degree of understanding of medication purpose.

Cognitive and emotional representations of illness were assessed with the Brief Illness Perception Questionnaire (Brief IPQ). Research has demonstrated the importance of illness representations in adaptation to chronic disease [16]. The first 8 questions of the Brief IPQ evaluate independent aspects of illness perception on a 0-10 Likert scale: consequences of illness, expected duration of illness, ability to personally control symptoms, ability of treatment to control symptoms, influence of illness on personal identity, concern about illness, understanding of illness, and emotional response to illness. The Brief IPQ has been validated in populations with diabetes, asthma, renal disease, and minor illnesses. It has been used in both adolescents and adults [14].

Self-care agency has been defined as "the power of an individual to engage in estimative and productive operations essential for self-care" [15]. This was assessed with the Exercise of Self-Care Agency (ESCA), a 35-item self-report questionnaire on a 5-point Likert scale. Total score ranges from 0-140. Higher scores have been associated with positive health behaviors [15]. The ESCA has been validated in multiple populations, including a cohort of American high school students [17].

Finally, 10 items assessed additional patient and disease characteristics that may mediate disease self-management. Demographics reported included age, gender and race. Disease-related factors included diagnosis and disease duration. Disease activity was assessed on a 0-10 Likert scale. Chart review was performed to confirm self-reported diagnosis and obtain additional disease-related data.

### Statistical Analysis

Summary statistics were used to describe all survey domains. Bivariate associations between self-reported medication adherence and other survey measures were assessed using the Student's t-test for normally distributed data, and the Wilcoxon rank-sum test for non-parametric data. Categorical associations were assessed using the Fisher exact test. In order to assess which self-care skills improve with age and which do not, independence in self-management tasks was compared between subjects age 13-16 and 17-20 using the chi-squared test. These age cut points were chosen in order to create groups of older and younger adolescents which could be applied across all measures, reflecting the developmental changes that occur in adolescence. Statistical analysis was performed using Stata version 11 for Macintosh (StataCorp LP, College Station, TX, USA).

## Results

Surveys were completed by 52 subjects (Table 1). Patients were 13-20 years old (mean 15.9) and 79% were female. Our sample was ethnically diverse: 50% White, 23% Asian, 8% Latino, 6% African American, and 13% unknown or other race. Diagnoses included juvenile idiopathic arthritis (44%), lupus (35%), dermatomyositis (8%), mixed connective tissue disease (6%), scleroderma (4%), idiopathic thrombocytopenic purpura (2%), and periodic fever syndrome (2%). Mean disease duration was 5.3 years (SD 4.0). Subjects took a mean of 3.3 medications (SD 2.0), and 58% were taking systemic corticosteroids. Mean self-reported disease activity on a 0-10 Likert scale was 4.1 (SD 2.6). While subjects age 13-16 were more likely to take methotrexate than their 17-20 year-old peers, there were no other statistically significant differences in characteristics between age groups.

**Table 1:** Demographics, disease characteristics and medications.

	N (%)	Mean (SD) or Median (Range)
Gender		
Male	11 (21%)	
Female	41 (79%)	
Mean age (SD)^#^		15.9 (2.1)
Race/Ethnicity		
Caucasian	26 (50%)	
Asian	12 (23%)	
Latino	4 (8%)	
African American	3 (6%)	
Other/Unknown	7 (13%)	
Diagnosis		
Juvenile Idiopathic Arthritis	23 (44%)	
Oligoarthritis	1 (2%)	
Rheumatoid factor positive polyarthritis	3 (6%)	
Rheumatoid factor negative polyarthritis	8 (15%)	
Systemic arthritis	8 (15%)	
Enthesitis-related arthritis	2 (4%)	
Psoriatic arthritis	1 (2%)	
Systemic Lupus Erythematosus	18 (35%)	
		
		
Juvenile Dermatomyositis	4 (8%)	
Mixed Connective Tissue Disease	3 (6%)	
Scleroderma	2 (4%)	
Idiopathic Thrombocytopenic Purpura	1 (2%)	
Periodic Fever Syndrome	1 (2%)	
Median Disease Duration (Range)^#^		5.3 (0-15)
Mean Self-Reported Disease Activity (SD)*		4.1 (2.6)
Medications		
Corticosteroids	30 (58%)	
Methotrexate or Leflunomide	24 (45%)	
Hydroxychloroquine	19 (37%)	
Biologic	15 (29%)	
Immunosuppressant^+^	13 (25%)	
Intravenous Immunoglobulin	2 (4%)	
Non-Steroidal Anti-Inflammatory Drug	2 (4%)	

^# ^Years.

* Disease activity reported on a 0-10 Likert scale, with 0 meaning "Not Active" and 10 meaning "Very Active."

^+ ^Immunosuppressants include cyclophosphamide, azathioprine, myophenolate mofetil, tacrolimus, and cyclosporine.

For the primary outcome measure - independent completion of health care management tasks (Table 2) - the majority of subjects know or carry emergency phone numbers (80%), have and know how to use a thermometer (68%), ask their doctor questions (65%), know symptoms of illness (63%), and take medications (63%). They were less likely to schedule their own medical appointments (17%), carry insurance information (21%), maintain a medical file (22%), get prescriptions filled (25%), or be aware of future changes in their own health insurance (26%). As compared to subjects age 13-16, 17-20 year olds showed increased ability to fill prescriptions, schedule appointments, arrange transportation to appointments, ask questions of their doctors, carry health insurance information, be aware of future changes in health insurance, and recognize symptoms of illness. However, older subjects were no more proficient than their younger peers at knowing medication names and purposes, taking medications as directed, keeping a calendar, maintaining a personal medical file, using a thermometer or knowing where to obtain contraception (Table 2).

**Table 2:** Independent Completion of Health Care Tasks.

	Age 13-16 (N = 31)	Age 17-21 (N = 21)	P
Medication Management			
Know medication names, purposes and side effects	15 (48%)	14 (67%)	0.26
Take medications as directed	17 (56%)	13 (62%)	0.78
Fill prescriptions	2 (6%)	11 (52%)	**<0.001**
Medical Appointments			
Schedule appointments	2 (6%)	6 (29%)	**0.05**
Keep a calendar of appointments	12 (39%)	10 (48%)	0.58
Arrange transportation to appointments	4 (13%)	13 (62%)	**0.001**
Prepare and ask questions of your doctor	16 (52%)	18 (86%)	**0.02**
Health Insurance and Information Management			
Carry health insurance information	3 (10%)	8 (38%)	**0.02**
Know about future changes in your health insurance	4 (13%)	9 (43%)	**0.02**
Maintain a personal medical file	4 (13%)	7 (33%)	0.10
Other Health Care Skills			
Have and know how to use a thermometer	20 (65%)	14 (67%)	1.0
Know symptoms of illness and when to call doctor	16 (52%)	17 (81%)	**0.04**
Know where to obtain contraception	11 (35%)	11 (52%)	0.26
Know or carry emergency phone numbers	23 (74%)	18 (85%)	0.50

For the secondary outcome measure - medication adherence - 54% reported perfect adherence, 40% reported 1-2 missed doses per week, and 6% reported missing >3 doses per week. Reasons for non-adherence included forgetfulness (54%), running out of medication (10%), and intentionally skipping doses (10%).

Medication concordance was assessed as a predictor of medication adherence. Among all subjects, mean concordance was 89% (range 25-100%) for knowing medication names, 78% (range 0-100%) for knowing the correct dosing regimen, and 54% (range 0-100%) for knowing the purpose of their medications. Concordance for medication purpose fell at two extremes: 35% of patients were able to describe the purpose of all of their medications and 30% were not able to state the purpose of any of their medications. The majority of patients use at least one memory aid to help them remember to take their mediations: 58% use a pill box; 6% use a timer; and 16% use other reminders, including cellular phone alarms, keeping bottles visible, and parental reminders. There was no statistically significant association between regimen knowledge or use of medication reminders and self-reported medication adherence (Table 3).

**Table 3:** Contribution of number of medications and medication knowledge to self-reported adherence.

	All patients (N = 52)**	Adherent^#^ (N = 21)	Nonadherent^#^ (N = 30)	P
Number of medications prescribed, mean (SD)	3.3 (2.0)	3.0 (2.5)	3.6 (1.4)	0.27
Number of patients using medication reminders, N (percent)	29 (58%)	11 (55%)	18 (60%)	0.73
Concordance for medication name, mean (range)*	89% (25-100%)	92% (44-100%)	87% (25-100%)	0.57
Concordance for medication dosing, mean (range)*	78% (0-100%)	82% (0-100%)	76% (25-100%)	0.39
Concordance for medication purpose, mean (range)*	54% (0-100%)	50% (0-100%)	56% (0-100%)	0.89

* Concordance was calculated as the number of correct patient responses divided by the total number of medications prescribed, reported as mean (range).

^# ^Adherence was defined as taking all prescribed doses of medication in a typical week.

** One patient did not report adherence.

Mean score on the Exercise of Self-Care Agency Scale was 73.2 (SD 16.9), as compared to 89.5 (SD 19.4) for a published report of high school students (P<0.001) [17]. There was a trend towards lower levels of self-care agency among subjects with higher self-reported adherence (P = 0.07). According to the Brief IPQ, most subjects believe medications can effectively treat their illness (7.2) and that they have moderate understanding of their illness (6.7). There were no significant associations between illness perception or other predictors and self-reported adherence.

## Discussion

Our findings provide insight into self-management proficiency and medication adherence among adolescents with chronic rheumatic disease. While this cohort showed good progress towards achieving independence in disease-management tasks in several domains, deficits were evident in other areas. Furthermore, older patients did not demonstrate an increase in proficiency in several areas as compared to their younger peers. Lack of improvement in self-management independence with increasing age suggests that adolescents are not mastering certain skills critical to self-management of chronic illness. Tasks that are most challenging for young patients involve interaction with adult-oriented systems and organizational skills, such as calling a medical office to schedule appointments, keeping a calendar, and maintaining a personal medical file.

While interventions to improve self-management have been shown to improve health outcomes and quality of life in both adults and children [18], these programs are not widely implemented. Data from several studies suggests that patient education interventions for CSHCN can be effective in improving health outcomes and decreasing loss to follow-up in the transition period [19]. Like many institutions, UCSF lacks a formal transition program for adolescents with chronic illness. Patients are expected to master disease self-management skills through parental coaching at home and physician encouragement as a part of routine care. However, our data suggest that this approach is not sufficient to achieve independence in all self-management skills in these older adolescents who are approaching transition.

Approximately half of our patients report imperfect adherence to medications, which is similar to other reported adherence rates among adolescents with chronic illness. The most common reason provided for missing medications was forgetfulness, a finding that has been reported previously [20]. Few subjects admit to intentionally skipping doses of medication, though subjects may be hesitant to disclose intentional non-adherence. Understanding the cause of medication non-adherence is important because while medication reminders may be effective for patients who forget to take their medications, they are unlikely to improve adherence if patients actively choose not to take their medications. Automated medication reminders have been studied extensively among adults with chronic disease, with some evidence of success [21].

With regard to predictors of self-reported medication adherence, accurately reporting the medication names and dosing regimens did not correlate with adherence. Interestingly, knowledge of medication purpose fell at two extremes, with the majority of patients demonstrating either very good understanding of the indications for their medications, or very poor understanding. It may be that some subjects have not been sufficiently engaged in their medical care or have not been willing to engage, and therefore lack understanding of the purposes of their medications. Other patients with pediatric-onset disease may have not be aware of the purpose of medications that they have taken since early childhood, when information about medications was directed towards the parent rather than the patient.

This study has several important limitations. Our results are derived from English-speaking patients at a single U.S. center and thus may not be generalizable to all practices. However, since UCSF is a Title V referral site, this study does draw from a diverse patient population representing Northern California. While subjects were recruited consecutively, our population may be biased towards more adherent patients, since those who did not appear for their appointments did not have the opportunity to participate in the study. Data on adherence and self-care practices were obtained via self-report, which is simple and cost-effective but may be subject to recall bias and social desirability bias [22]. In addition, the adherence measures used were developed by the authors and not tested prior to use. Our sample size was also small; however, rheumatic diseases in children are relatively rare. Finally, quantitative analysis may not be able to fully and accurately describe complex behavior around adherence.

## Conclusions

Transition to adulthood is an exciting and a challenging time, when adolescents and young adults must complete many educational, social, and vocational tasks. For adolescents with a diagnosis of lifelong chronic illness, mastering disease self-management is a critical part of this transition. This study suggests that the current standard of care is inadequate to prepare adolescents to successfully manage their disease in adulthood. Further work is needed to assess deficits in self-care skills in a broader population of adolescents with rheumatic disease to ensure generalizability of the current findings, which can then be applied to the development of targeted self-care improvement programs. Interventions to improve self-management should be integrated into standardized transition readiness procedures that address both the self-management and health care systems aspects of transition to adult care, and ultimately lead to improved health outcomes.

## Competing interests

The authors declare that they have no competing interests.

## Authors' contributions

EFL performed the statistical analysis and drafted the manuscript. AOH conceived the study, designed the survey, and participated in data interpretation and manuscript compilation. MAA, EHY and MJO participated in data interpretation and manuscript compilation. EVS conceived the study, and participated in its design and coordination and helped to draft the manuscript. All authors read and approved the final manuscript.
